# Development of a novel multifocal lens using a polarization directed flat lens: possible candidate for a multifocal intraocular lens

**DOI:** 10.1186/s12886-021-02191-z

**Published:** 2021-12-27

**Authors:** Kyung-Sun Na, Chang Su Lee, Da Ran Kim, Seok Ho Song, Soo Yeon Cho, Eun Chul Kim, Hyun Seung Kim, Ho Sik Hwang

**Affiliations:** 1 Department of Ophthalmology, College of Medicine, The Catholic University of Korea, Seoul, Republic of Korea, 505, Banpo-dong, Seocho-gu, Seoul, 06591 Republic of Korea; 2 Department of Electrical and Electronic Engineering, The University of Suwon, Hwaseong, South Korea; 3 Department of Physics, Hanyang University, Seoul, South Korea

**Keywords:** Multifocal lens, Polarization-directed flat: intraocular lens

## Abstract

**Background:**

A polarization-directed flat (PDF) lens acts as a converging lens with a focal length (f) > 0 and a diverging lens with f < 0, depending on the polarization state of the incidental light. To produce a multifocal lens with two focal lengths, a PDF and a converging lens having shorter focal length were combined. In this study, we tested a bifocal PDF to determine its potential as a new multifocal intraocular lens (IOL).

**Methods:**

Constructed a multifocal lens with a PDF lens (f = +/− 100 mm) and a converging lens (f = + 25 mm). In an optical bench test, we measured the defocus curve to test the multifocal function. The multifocal function and optical quality of the lens in various situations were tested. An Early Treatment Diabetic Retinopathy Study (ETDRS) chart as a near target and a building as a distant target were photographed using a digital single-lens reflex (DSLR) camera. Both lenses (multifocal and monofocal) were tested under the same conditions.

**Results:**

For the 0 D and − 20 D focal points, the multifocal lens showed sharp images in the optical bench test. In the DSLR test using the multifocal lens, the building appeared slightly blurry compared with the results using the monofocal lens. With the multifocal lens, the ETDRS chart’s images became blurry as the ETDRS chart’s distance decreased, but became very clear again at a certain position.

**Conclusions:**

We confirmed the multifocal function of the multifocal lens using a PDF lens. This lens can be used as a multifocal IOL in the future.

**Supplementary Information:**

The online version contains supplementary material available at 10.1186/s12886-021-02191-z.

## Background

The use of multifocal intraocular lenses (IOLs) in cataract surgery continues to increase. Diffractive multifocal IOLs and refractive multifocal IOLs are predominant among the currently applied multifocal lenses [1]. We recently developed a new multifocal lens by combining a polarization-directed flat (PDF) lens and a converging lens.

PDF lenses (geometric phase lenses) utilize a spatial-varying phase change generated through a closed path in the polarization parameter space, which is commonly realized using a patterned liquid crystal (LC) half-wave plate [2]. The orientation of LC molecules at each spatial point on the lens surface determines the local phase change. Because of their continuous phase profile and flat geometry, PDF lenses provide higher optical quality and less stray light in comparison with Fresnel phase lenses.

**Fig. 1:**
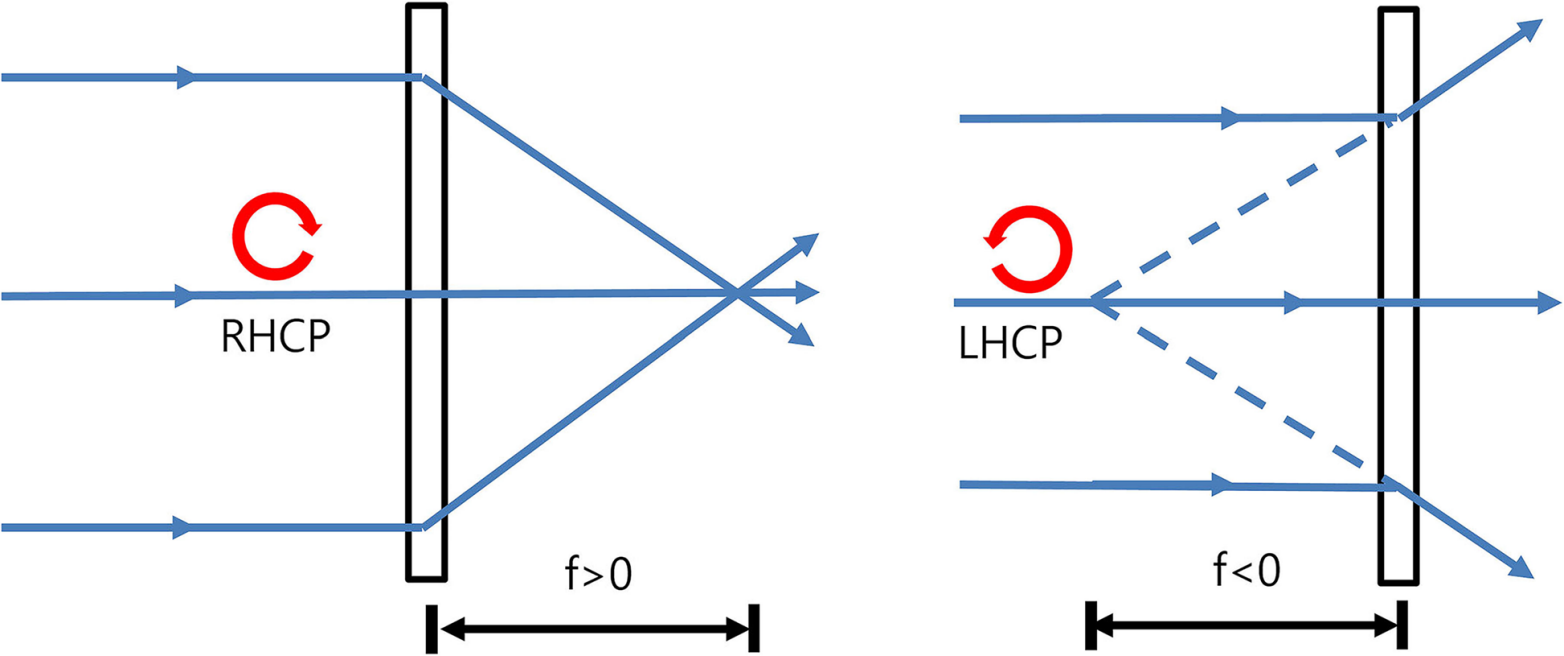
Polarization-directed flat (PDF) lens. When the incidental light is right-handed circular polarization (RHCP), the focal length of the PDF lens is positive. On the other hand, left-handed circular polarization (LHCP) generates a negative focal length. That is, the PDF lens acts as a converging lens with focal length (f) > 0 and a diverging lens with f < 0, depending on the polarization state of the incidental light

**Fig. 2:**
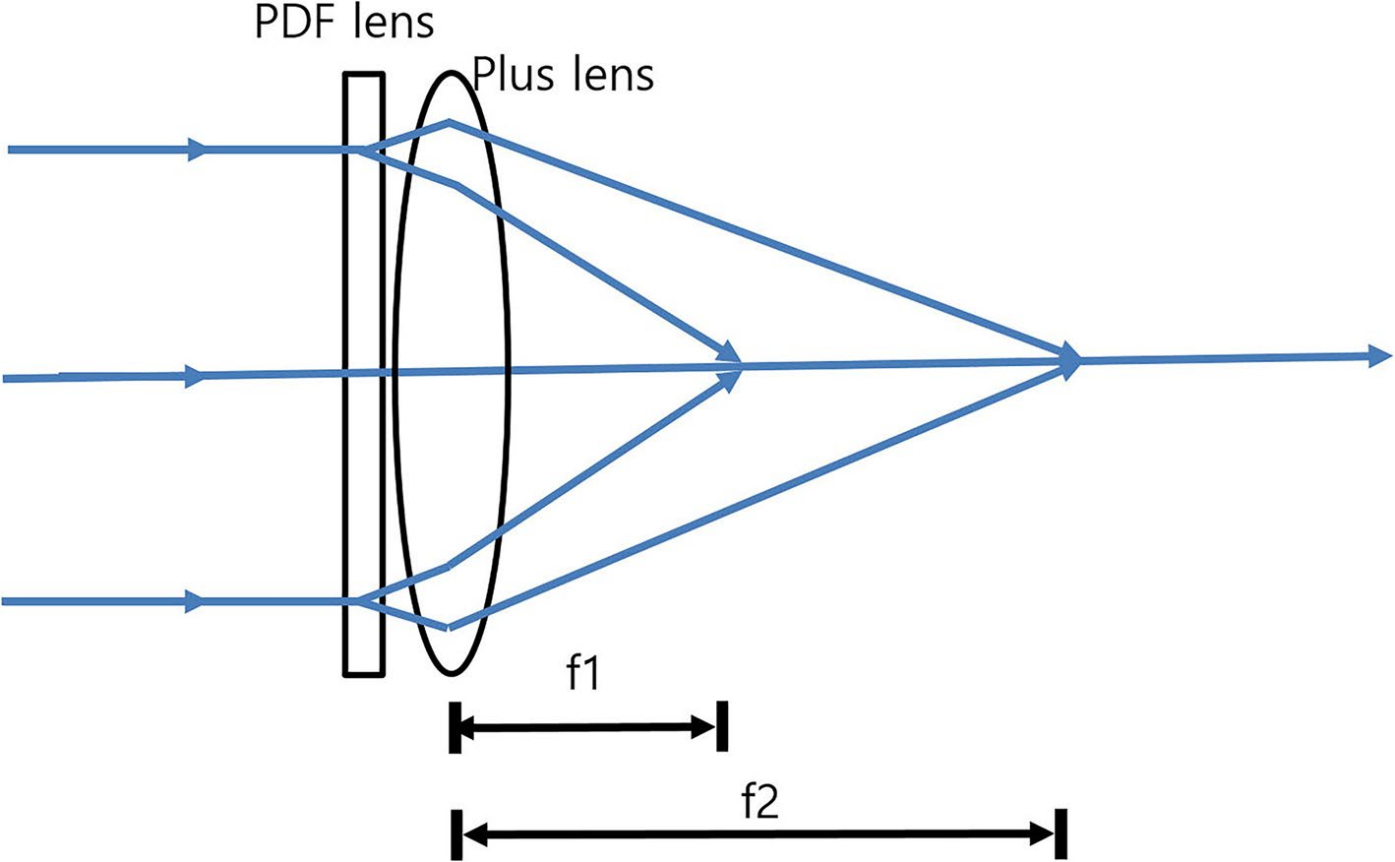
The principle of the new multifocal lens. By combining a polarization-directed flat (PDF) lens and a converging lens with a shorter focal length, a multifocal lens with 2 focal lengths can be constructed

A PDF lens consists of a thin flat film in which photo-aligned geometric phase holograms are recorded [3–6]. The phase holograms cause phase shifts that depend on the polarization states of the incidental light illuminated onto the PDF lenses [3–6]. When the incidental light is right-handed circular polarization (RHCP), the focal length of the PDF lenses is positive. On the other hand, left-handed circular polarization(LHCP) generates a negative focal length [6]. That is, the PDF lens acts as both a converging lens with a focal length (f) > 0 and a diverging lens with f < 0, depending on the polarization state of the incidental light (Fig. 1). Combining the PDF lens with a converging lens having a shorter focal length creates a bifocality, f1 and f2 (Fig. 2). For example, if a +/− 100 mm (+/− 10 D) PDF lens is combined with a + 25 mm (+ 40 D) converging lens, the resulting lens will be a bifocal lens with + 30 D/+ 50 D. According to the manufacturer’s data for the PDF lens we used, the lens serves as a converging lens when f > 0 for 50`% incidental light and a diverging lens when f < 0 for 50% incidental light, not only for circularly polarized light but also for unpolarized light (https://www.edmundoptics.com/globalassets/documents/polarization-directed-flat-lens-overview.pdf).

Although there many technical reports on PDF lenses, [3–6] this is the first report, to our knowledge, describing a new multifocal lens that combines a PDF lens and a converging lens. In the present study, we tested the multifocal function and optical quality of our multifocal lens to determine its potential as a new multifocal IOL.

## Methods

### Optical bench test

**Fig. 3:**
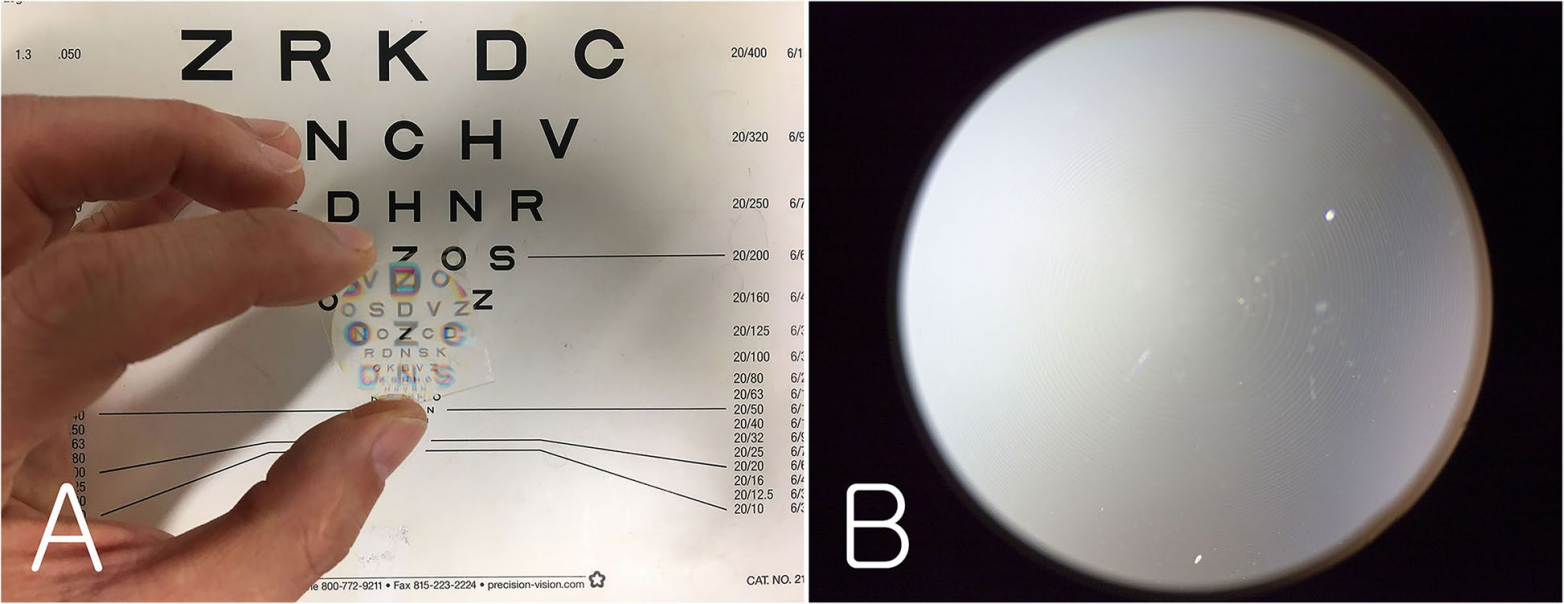
The polarization-directed flat lens used in this study. **A** focal length +/− 100 mm, diameter 25 mm, and thickness 0.45 mm; **B** Concentric rings could be seen under a stereomicroscope (X20)

The PDF lens (Edmund Optics, Barrington, NJ) used in the optical bench test had a focal length of +/− 100 mm, a diameter of 25 mm, and a thickness of 0.45 mm (Fig. 3). Concentric rings were observed under a stereomicroscope (X20, SM-4TZ-30WY-16 M3, Amscope, Irvine, CA). The converging lens used to make the multifocal lens was an achromatic lens with a focal length of + 25 mm, diameter of 12.7 mm, and thickness of 7.0 mm (Thorlabs Inc., Newton, NJ). The 2 lenses were combined to create a multifocal lens. The experimental setting for measuring the multifocal function and optical quality of this lens was as described below.

**Fig. 4:**
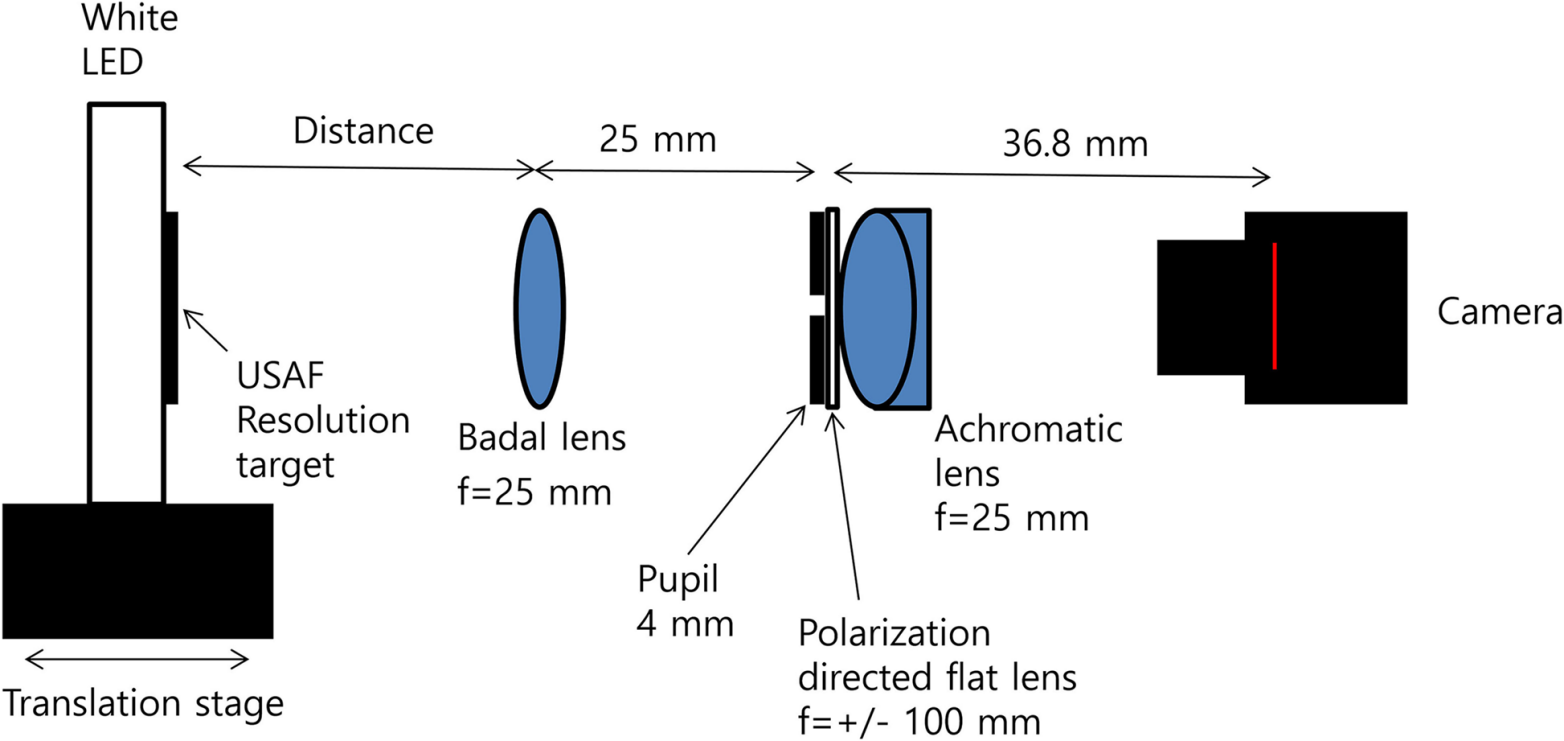
Optical bench test setting. A white light-emitting diode (LED; 3-3/4 in. LED Square Plate for Microscopes, AmScope), USAF 1951 resolution target (2“ x 2” Negative 1951 USAF Hi-Resolution Target, Edmund Optics), Badal lens (achromatic lens, focal length + 25 mm; Thorlabs Inc.), 4 mm-diameter pupil, PDF lens (focal length +/− 100 mm), achromatic lens (focal length + 25 mm), and charge-coupled device (CCD) camera (acA1600-20uc, Basler, Ahrensburg, Germany) were arranged linearly

A white light-emitting diode (LED; 3-3/4 in. LED Square Plate for Microscopes, AmScope), USAF 1951 resolution target (2“ x 2” Negative 1951 USAF Hi-Resolution Target, Edmund Optics), Badal lens (achromatic lens, focal length + 25 mm; Thorlabs Inc.), 4 mm-diameter pupil, PDF lens (focal length +/− 100 mm), achromatic lens (focal length + 25 mm), and charge-coupled device (CCD) camera (acA1600-20uc, Basler, Ahrensburg, Germany) were arranged linearly (Fig. 4). From the Badal lens to the CCD camera, all items were connected to a 30-mm cage system to eliminate the need for additional alignment. The LED and USAF resolution target were fixed to an XYZ translation stage (Thorlabs Inc.). While viewing the image on a monitor connected to the camera, the USAF resolution target was aligned at the axis of the 30-mm cage system by moving the stage.

### Monofocal lens (achromatic lens only)

USAF resolution targets were photographed with only an LED, USAF 1951 resolution target, Badal lens (achromatic lens, focal length + 25 mm), 4-mm diameter pupil, achromatic lens (focal length + 31.8 mm; Newport Co., Irvine, CA), and CCD camera. The distance between the Badal lens with a focal length of + 25 mm and the pupil was set to 25 mm. The distance between the achromatic lens with a focal length of + 31.8 mm and camera sensor was set to 31.8 mm. The USAF target with an LED started at 30 mm (+ 8 D) from the Badal lens. The USAF target was brought closer to the Badal lens until the distance between them was 20 mm (− 8 D). In this Badal type configuration, the magnification of the USAF target will remain constant with this motion. Photos were obtained every 0.5 mm during the approach. We moved the USAF with LED finely using a XYZ micrometer translation stage (Thorlabs Ins.).

**Fig. 5:**
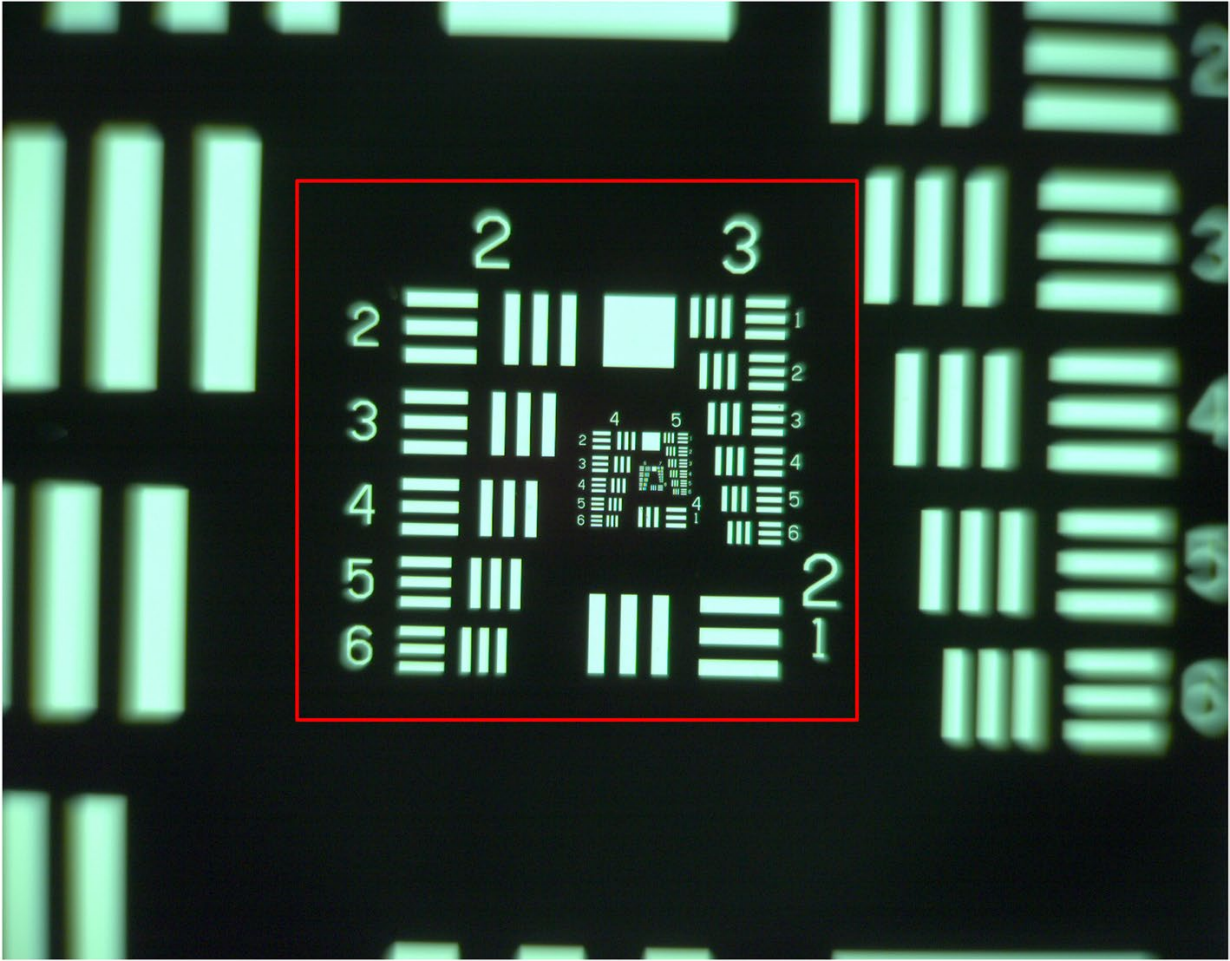
USAF 1951 resolution target. As a reference template image for obtaining the cross-correlation coefficient, a middle rectangular area was selected and analyzed from a clear USAF resolution image (from group 2 to 7 elements)

To quantify the quality of each image of the resolution target, we computed their cross-correlation coefficients. In general, a cross-correlation coefficient is used to quantify the similarity between 2 images [7–9]. To quantify the sharpness of each image, the similarity between the 2 images was compared using the 0 D defocus (sharpest) image as a reference. Therefore, a cross-correlation coefficient that quantifies similarity can be used. As a reference template image for obtaining the cross-correlation coefficient, a middle rectangular area was selected and analyzed from a clear USAF resolution image (from group 2 to 7 elements; Fig. 5). The cross-correlation coefficient between the test image f(x, y), including the blurred reference image and the sharp reference image t(x, y), can be obtained as follows.

1 $$\rho \left(u,v\right)=\frac{\sum_{x,y}\left[f\left(x,y\right)-\overline{f_{u,v}}\right]\left[t\left(x-u,y-v\right)-\overline{t}\right]}{{\left\{{\sum}_{x,y}{\left[f\left(x,y\right)-\overline{f_{u,v}}\right]}^2{\sum}_{x,y}{\left[t\left(x-u,y-v\right)-\overline{t}\right]}^2\right\}}^{1/2}}$$

Here, $\overline{t}$ is the mean value of the reference image; $\overline{f_{u,v}}$ is the center of the coordinates (u, v) of the test image and the mean value of the same size image as the reference image.

To calculate the cross-correlation coefficient from the coordinates (u, v) of the test image, the center of the reference image was centered on these coordinates, and the pixels f(u, v) and t(u, v) subtracted by the mean value were multiplied pixel by pixel and normalized by dividing by its magnitude. The cross-correlation ranged from − 1 to + 1, and + 1 indicated that the image was the same as the best query reference image. The cross-correlation indicated that the quality of the image decreased as the value of it decreased. The value of − 1 indicated that the black and white pixels of the 2 images were inverted. When it was overlaid on the test image and scanned from the first row to the right column, the cross-correlation coefficient matrix from the first row to the last row was obtained as in Eq. (1). Among the coefficients of the matrix, the peak value was generally present in one place, and this value was the optimal cross-correlation coefficient. In addition, the best query-like image was obtained from the coordinate u and v information. The experimental results were as follows. The size of the actual test image was 2592 × 2048 pixels and the size of the reference image extracted from it was 1077 × 1012 pixels (Fig. 5).

The cross-correlation coefficient of the acquired test image should be calculated with the image of the same magnification as the reference image. To this end, the magnification of the test image was calculated based on the number of pixels corresponding to the height of the rectangular border area, excluding the numbers of reference images. According to this magnification, the size of the test image was obtained again by a third-order polynomial interpolation method. In obtaining the cross-correlation coefficient with the test image considering magnification, it was possible to obtain the cross-correlation coefficient from most test images. In the scaled image, the partial image at the position where the peak of the cross-correlation occurs was displayed as a rectangular box. If the test image was severely blurred, however, it was not possible to find a matching region with only the maximum value of the correlation coefficient. In some test images, the cross-correlation coefficients were obtained manually. A defocus curve was obtained by measuring the cross-correlation coefficient with respect to the defocus point, using MATLAB software (MathWorks; Natick, MA).

### Multifocal lens (PDF lens + achromatic lens)

USAF resolution targets were photographed with a white LED, USAF 1951 resolution, Badal lens (achromatic, focal length + 25 mm), 4 mm-diameter pupil, PDF lens (focal length +/− 100 mm), achromatic lens (focal length + 25 mm), and CCD camera. A polarizer was not used. The distance between the Badal lens with a focal length of 25 mm and the pupil was set to 25 mm. The PDF lens was fixed with tape in front of the lens mount of the achromatic lens (near the convex surface of the achromatic lens) and confirmed to be centered. For exact centration, we fixed the PDF lens on a 30-mm cage plate (Thorlabs Inc.) for the achromatic lens mount so that all four sides of the PDF lens contacted a center-located bore margin of it (Supplemental Fig. 1).

The PDF lens almost contacted the convex surface of the achromatic lens. The distance between the multifocal lens and the camera sensor was adjusted so that the longer focal point of the multifocal lens was located on the camera sensor (theoretically 36.8 mm between PDF lens and camera sensor). The USAF target with the LED was positioned closer to the Badal lens (f = 25 mm) in 0.5-mm increments from 29.88 mm (+ 7.8 D) to 6.88 mm (− 29.0 D). We obtained 16 photos during the approach. The USAF with LED was precisely moved using an XYZ micrometer translation stage (Thorlabs Ins.). We obtained a defocus curve that shows the change in the cross-correlation coefficient with respect to the defocus point.

At this time, the 0 D defocus (sharpest) image obtained with the above monofocal lens (achromatic lens only) was used as a reference image for calculating the cross-correlation coefficient.

During the experiment, the LED brightness and camera settings (exposure time, International Organization for Standardization [ISO], gamma value, white balance, etc.) were not changed.

We took photos at distant (0 D) and near focus (− 20 D) after adding blue (395-480 nm; Edmund Optics), green (520-550 nm; Edmund Optics), and red filters (cut on 600 nm; Edmund Optics) in front of USAF target to check whether the 50/50 division of light was valid at any wavelength across the visible spectrum for this multifocal lens. We calculated the cross-correlation coefficient of the images. The 0 D defocus (sharpest) image obtained with the above monofocal lens (achromatic lens only) was used as a reference image for calculating the cross-correlation coefficients.

## Digital single-lens reflex camera test

**Fig. 6:**
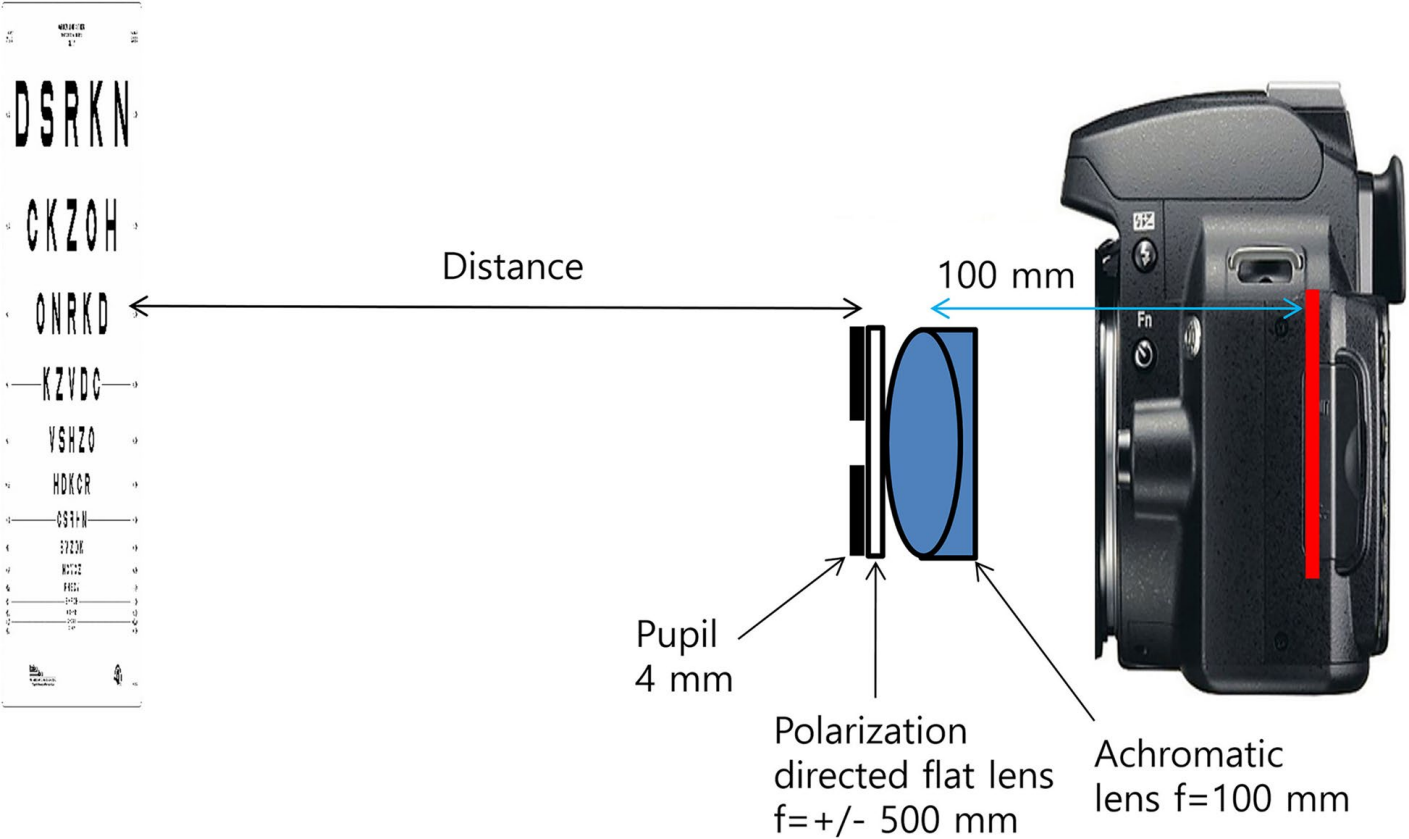
Digital single-lens reflex (DSLR) camera test setting. To test the multifocal lens, a 4-mm pupil, focal length +/− 500 mm (+/− 2 D) PDF lens (provided by Professor Seok Ho Song), achromatic lens (focal length 100 mm), and a DSLR camera were used

The multifocal function and optical quality of a multifocal lens (PDF lens + converging lens) in various conditions were tested using a digital single-lens reflex (DSLR) camera (D850, Nikon, Tokyo, Japan; Fig. 6).

To test monofocal lens, the system was made of a 4-mm pupil, achromatic lens (focal length + 100 mm) (Thorlabs Inc.), and DSLR camera. They all were connected to a 30-mm cage system to eliminate the need for additional alignment. Focus was achieved by adjusting the distance between the camera and the achromatic lens. When a far object more than 6 m away appeared clear, the achromatic lens was fixed at the cage system. After focusing, the ambient light was shielded with black tape, and objects at far and near distances were photographed or recorded as videos.

**Fig. 7:**
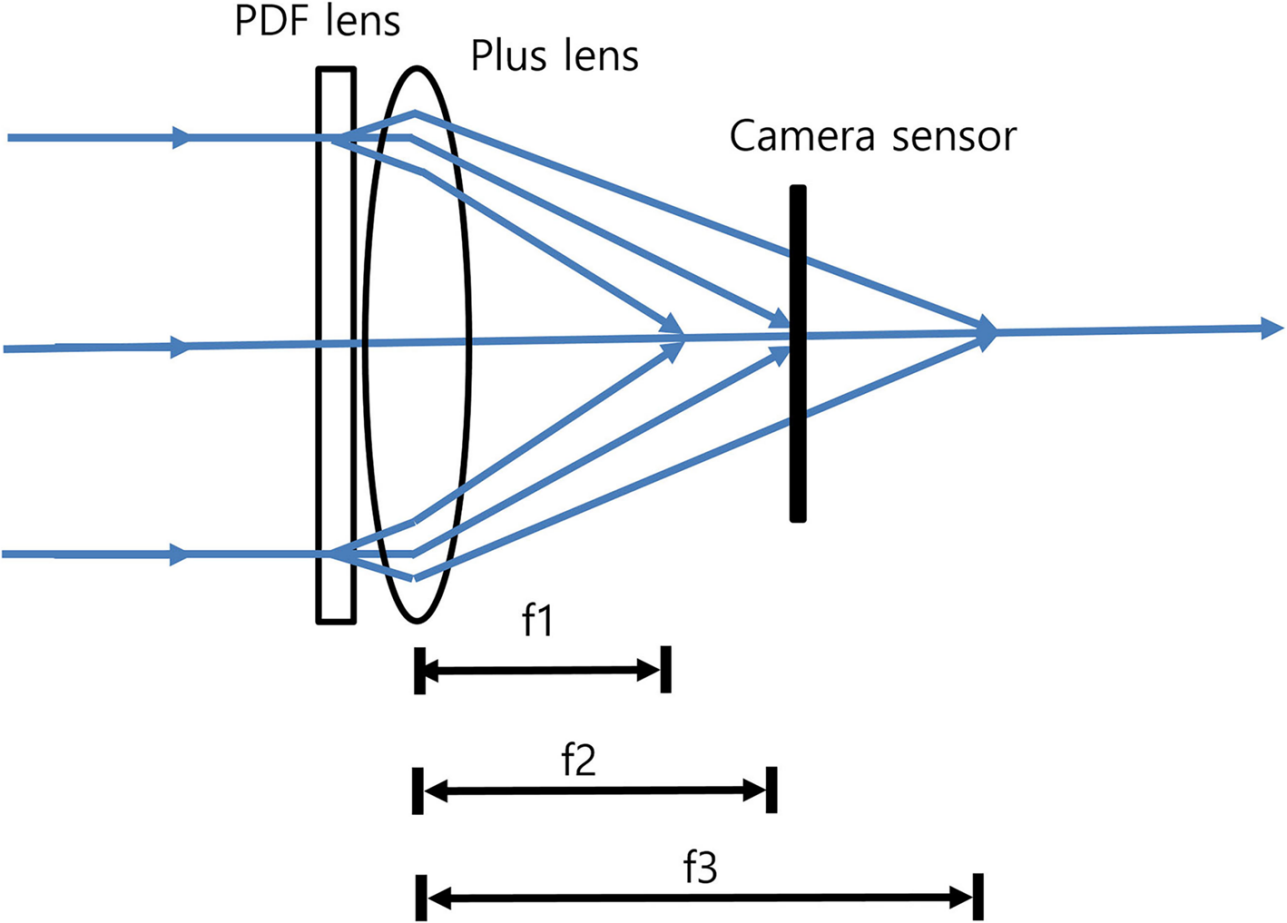
Polarization-directed flat (PDF) lens used for digital single-lens reflex (DSLR) camera test. This PDF lens has a focal length of +/− 500 mm (+/− 2 D) depending on the polarization state of the incidental light and allows some of the light to pass through without refraction. Combining this PDF lens with an achromatic lens (focal length 100 mm, + 10 D) results in a trifocal lens (+ 8, 10, 12 D)

To test the multifocal lens, a 4-mm pupil, focal length +/− 500 mm (+/− 2 D) PDF lens (provided by Professor Seok Ho Song), achromatic lens (focal length 100 mm), and a DSLR camera were used. This PDF lens has a focal length of +/− 500 mm (+/− 2 D) depending on the polarization state of the incidental light and allows some of the light to pass through without refraction. Combining this PDF lens with an achromatic lens (focal length 100 mm, + 10 D) results in a trifocal lens (+ 8, 10, 12 D) (Fig. 7). The PDF lens was fixed with tape in front of the lens mount of the achromatic lens (near the convex surface of the achromatic lens) and it was confirmed to be centered. A polarizer was not used. Focus was achieved by adjusting the distance between the camera and the multifocal lens. At this time, the multifocal lens was positioned so that the second focal point (+ 10 D) of the multifocal lens was located at the sensor of the camera. After focusing, the ambient light was shielded with black tape, and objects at far and near distances were photographed or recorded as videos.

### Far distance

During the day, a building was photographed with a DSLR camera, and this was repeated with a monofocal lens and a multifocal lens. Using a tripod, we attempted to capture the same view with both the monofocal and multifocal lenses.

### Near distance

To test multifocal function, the Early Treatment Diabetic Retinopathy Study (ETDRS) chart (ETDRS 2000 Series chart “2” [Precision Vision, La Salle, IL]) was used as the near target. The ETDRS chart started at 660 mm from the iris. The ETDRS chart was brought closer to the DSLR camera until it reached 30 mm, i.e., the distance between the iris and the ETDRS chart was 30 mm; Fig. 6.) The approach toward the camera was recorded as a video. General stand lighting was used. This was repeated for the monofocal lens and the multifocal lens.

### Far and near distance

To test the multifocal function in the daytime, the ETDRS chart at near distance was recorded as a video right after recording a building. This was repeated for the monofocal lens and the multifocal lens.

## Results

### Optical bench test

**Fig. 8:**
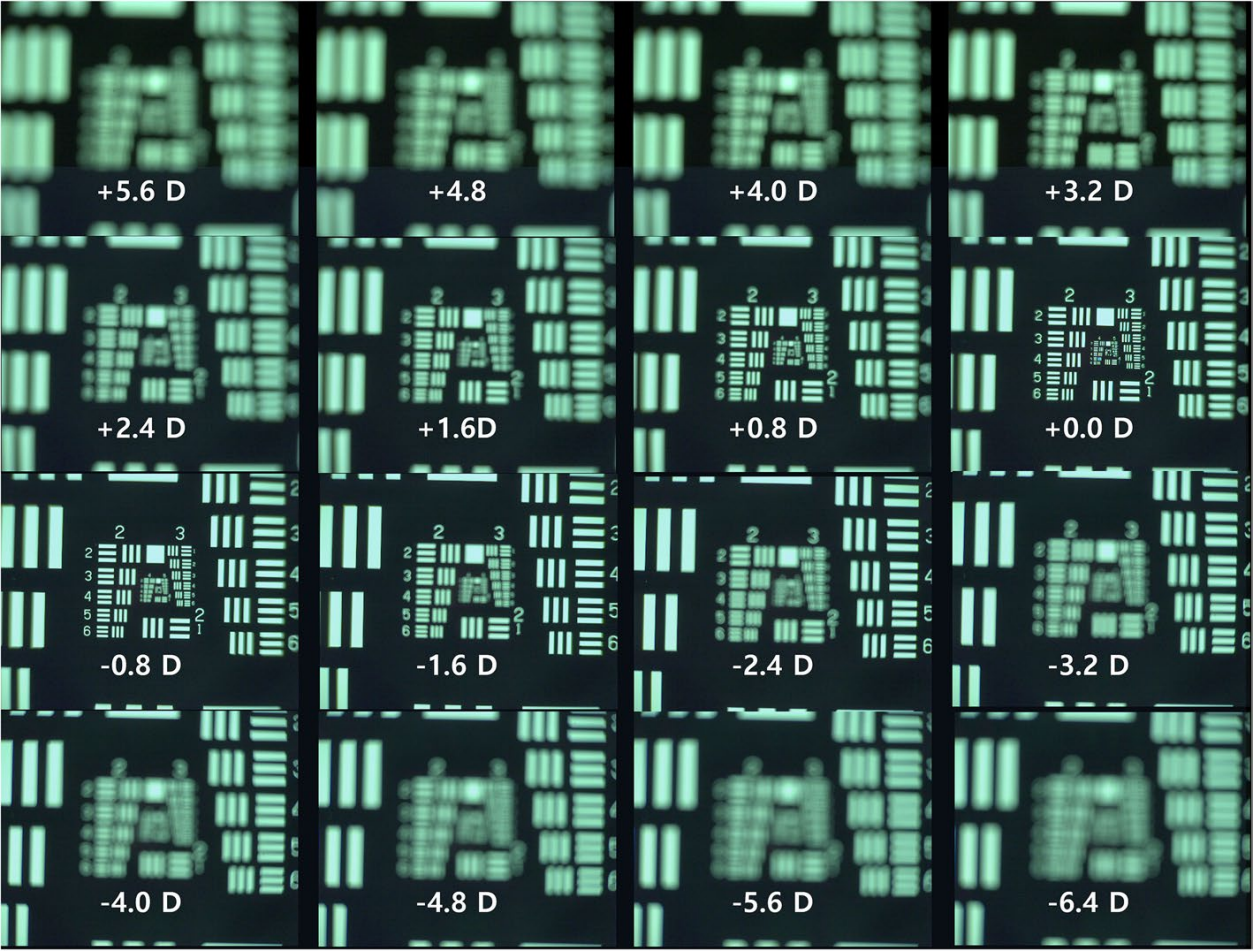
Optical bench test (monofocal lens). This figure shows USAF resolution of target images taken with a monofocal lens. With the monofocal lens, the image was sharpest (in focus) when the distance between the USAF target and Badal lens was 25 mm (0 D), but the images quickly became blurry when it was out of focus

**Fig. 9:**
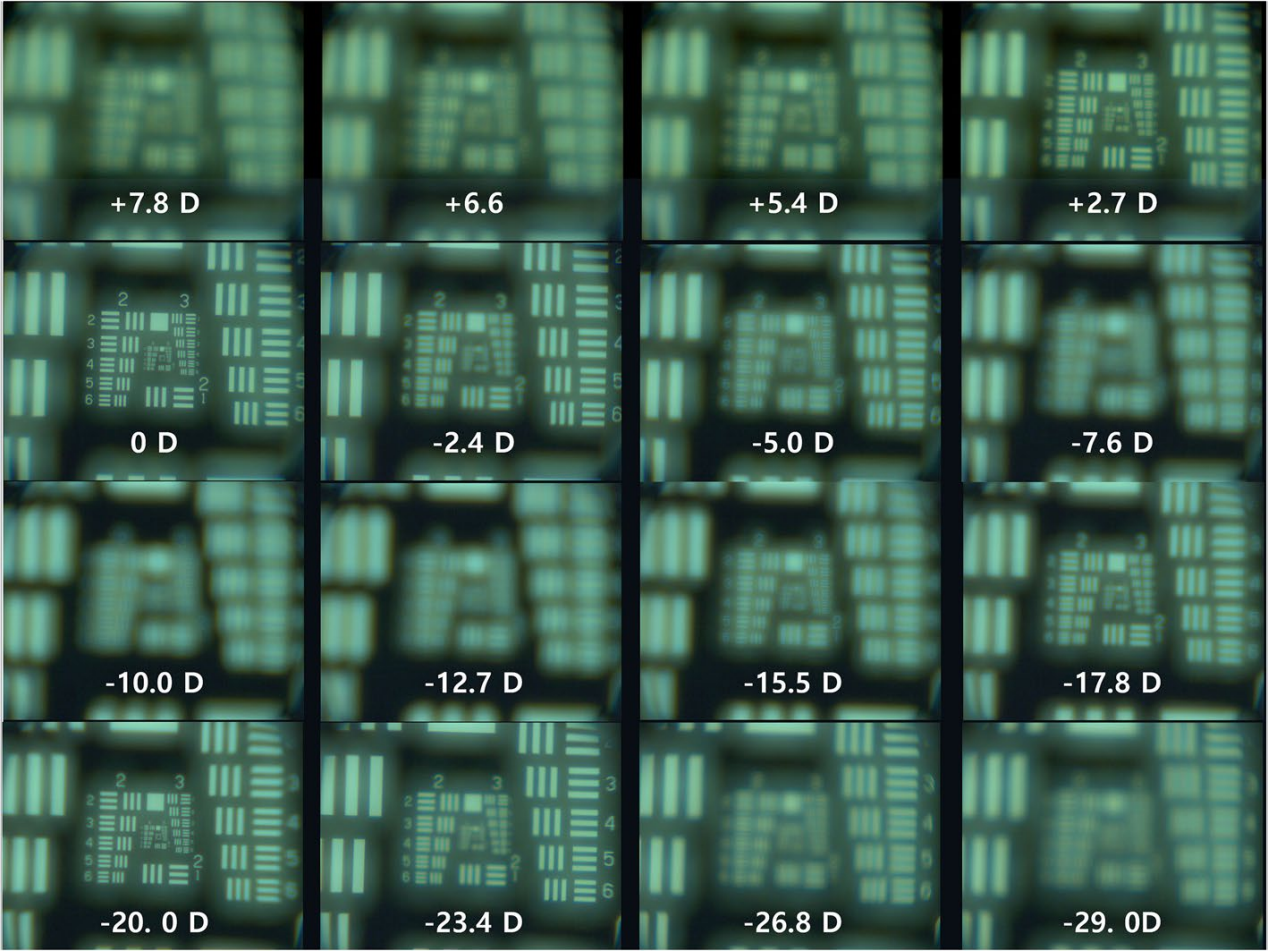
Optical bench test (multifocal lens). With the multifocal lens, when the distance between the USAF resolution target and Badal lens was 25 mm (0 D), the USAF resolution target image was clear. As the distance decreased, the image became blurry, but the image became clear again when the distance between the USAF resolution target and Badal lens was 12.5 mm (− 20 D)

**Fig. 10:**
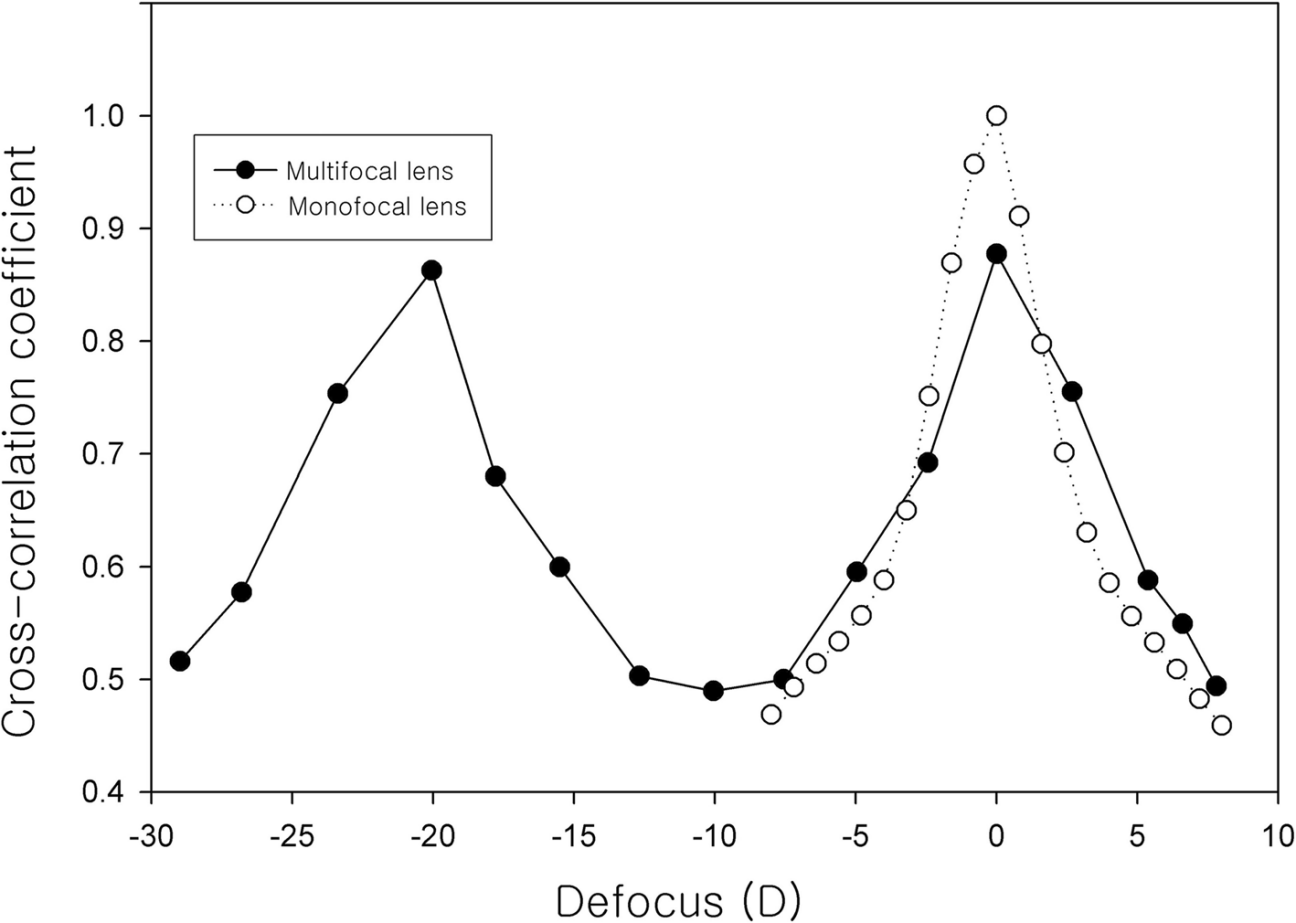
Cross-correlation coefficient of monofocal and multifocal lens. The cross-correlation coefficient curve of monofocal lens showed a very high and narrow peak centered at 0 D. The cross-correlation coefficient curve of multifocal lens showed the profile of a bifocal lens showing two peaks, at 0 D (cross-correlation coefficient: 0.878) and − 20 D (cross-correlation coefficient: 0.863)

Figure 8 shows USAF resolution target images taken with a monofocal lens, and Fig. 9 shows USAF resolution target images taken with a multifocal lens. With the monofocal lens, the image was sharpest (in focus) when the distance between the USAF target and Badal lens was 25 mm (0 D), but the images quickly became blurry when it was out of focus (Fig. 8). The image at 0 D was used as a reference template for cross-correlation coefficient calculation. The cross-correlation coefficient curve showed a very high and narrow peak centered at 0 D (Fig. 10). The cross-correlation coefficient at 0 D was 1.0 by definition. Chromatic aberration was rarely observed.

With the multifocal lens, when the distance between the USAF resolution target and Badal lens was 25 mm (0 D), the USAF resolution target image was clear. As the distance decreased, the image became blurry, but the image became clear again when the distance between the USAF resolution target and Badal lens was 12.5 mm (− 20 D) (Fig. 9). At most distances, chromatic aberration was observed at the edge of the letters compared with the monofocal lens. The image at 0 D of monofocal was used as a reference template for cross-correlation coefficient calculation. The cross-correlation coefficient curve showed the profile of a bifocal lens showing two peaks, at 0 D (cross-correlation coefficient: 0.878) and − 20 D (cross-correlation coefficient: 0.863; Fig. 10). The cross-correlation coefficients were all less than 1.0, however, indicating that they were blurrier than the in-focus image (0 D) obtained with the monofocal lens.

**Fig. 11:**
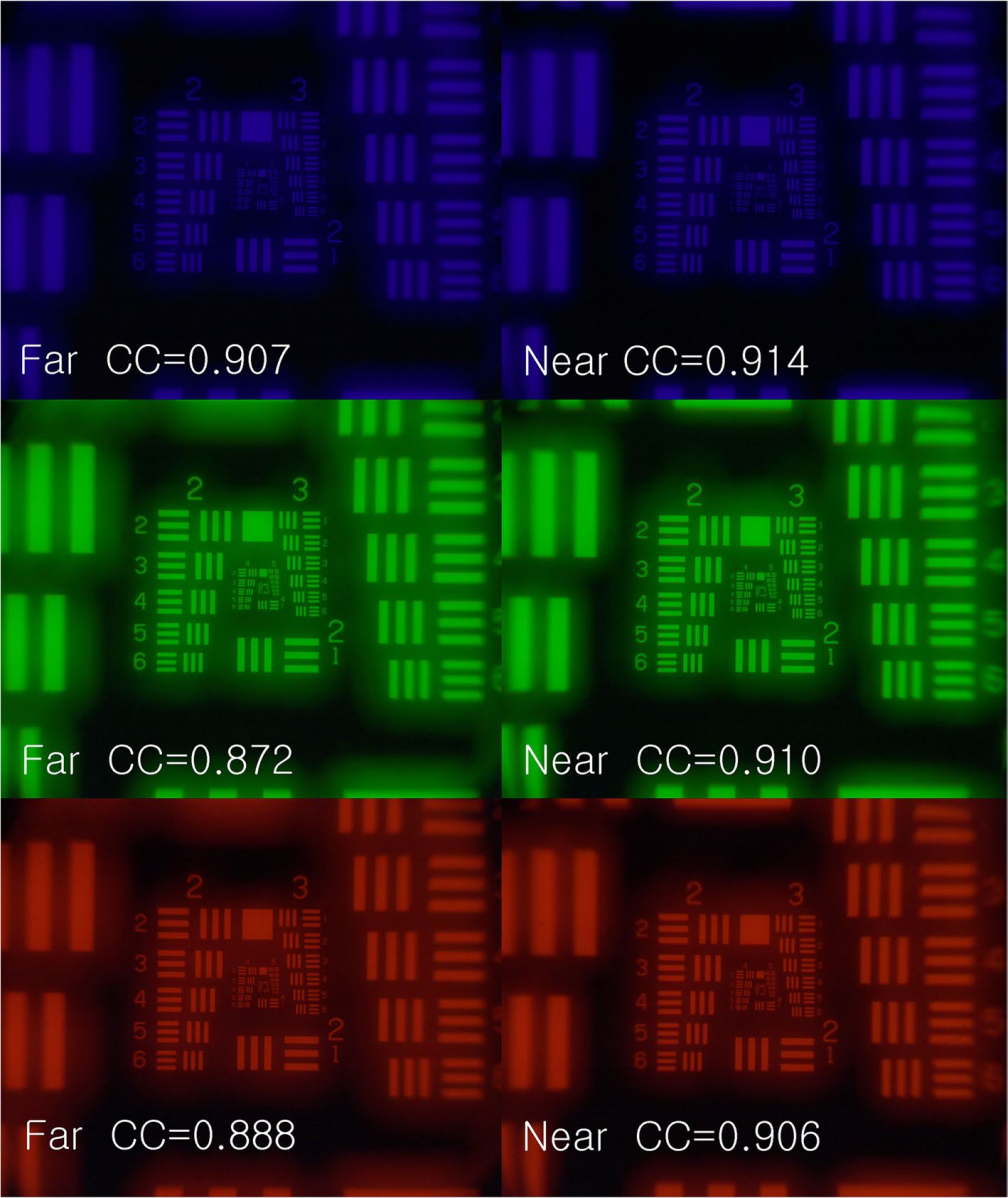
The images at near and distant focus taken with blue (top), green (middle), and red (bottom) filters. In quantitative analyses, the cross-correlation coefficient was 0.907 at distant focus and 0.914 at near focus with a blue filter. The cross-correlation coefficient was 0.872 at distant focus and 0.910 at near focus with a green filter. The cross-correlation coefficient was 0.888 at distant focus and 0.906 at near focus with a red filter

Figure 11 shows the images at near and distant focus taken with blue, green, and red filters. In quantitative analyses, the cross-correlation coefficient was 0.907 at distant focus and 0.914 at near focus with a blue filter. The cross-correlation coefficient was 0.872 at distant focus and 0.910 at near focus with a green filter. The cross-correlation coefficient was 0.888 at distant focus and 0.906 at near focus with a red filter. These findings suggest that a 50/50 division of light is valid at all wavelengths across the visible spectrum for this multifocal lens.

### DSLR camera test

#### Far distance

**Fig. 12:**
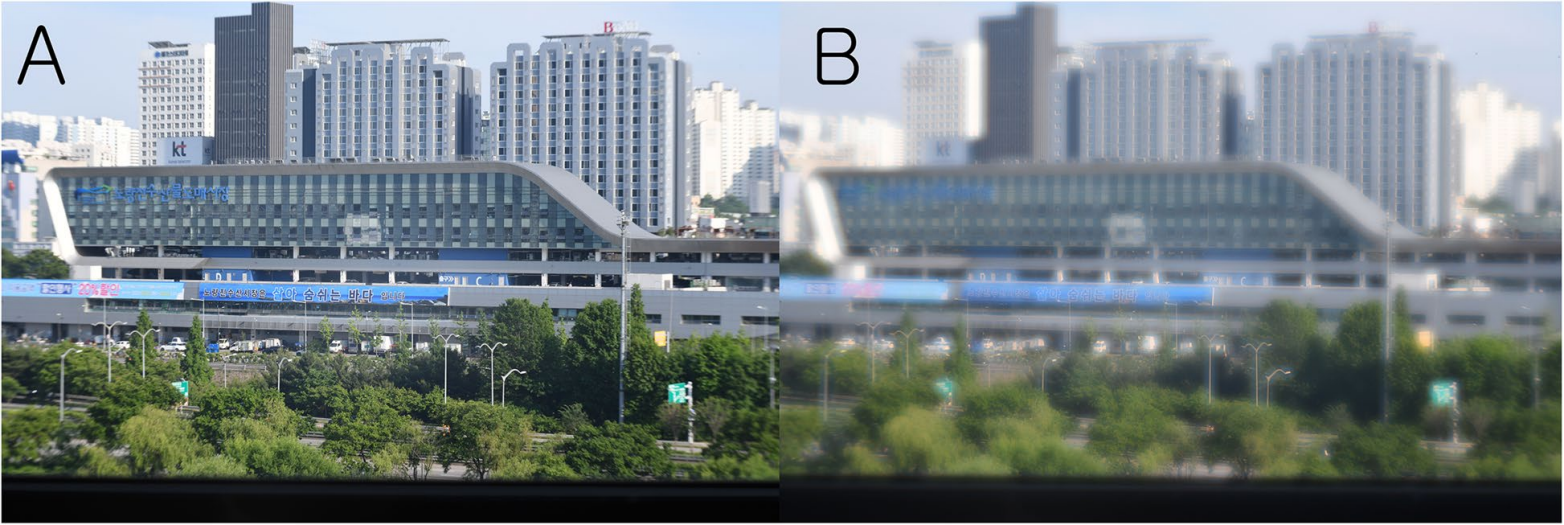
Digital single-lens reflex (DSLR) camera test (Far distance, day). This figure shows a far-distant building photograph taken with a monofocal lens (**A**) and a multifocal lens (**B**). With the monofocal lens, the building appeared very clear. With the multifocal lens, the building appeared slightly blurry compared with the results from the monofocal lens. This was especially true around bright objects. Chromatic aberration was observed around the bright objects

Figure 12 shows a far-distant building photograph taken with a monofocal lens (A) and a multifocal lens (B). When focusing at a far distance with a monofocal lens, the distance between the lens and the DSLR camera sensor was 100 mm. With the monofocal lens, the building appeared very clear. No chromatic aberration was observed (Fig. 12A).

When the second focal point (+ 10 D) of the multifocal lens was located at the sensor of the camera, the distance between the PDF lens and the DSLR camera sensor was 100 mm. With the multifocal lens, the building appeared slightly blurry (Fig. 12B) compared with the results from the monofocal lens. This was especially true around bright objects. Chromatic aberration was observed around the bright objects.

#### Near distance

**Fig. 13:**
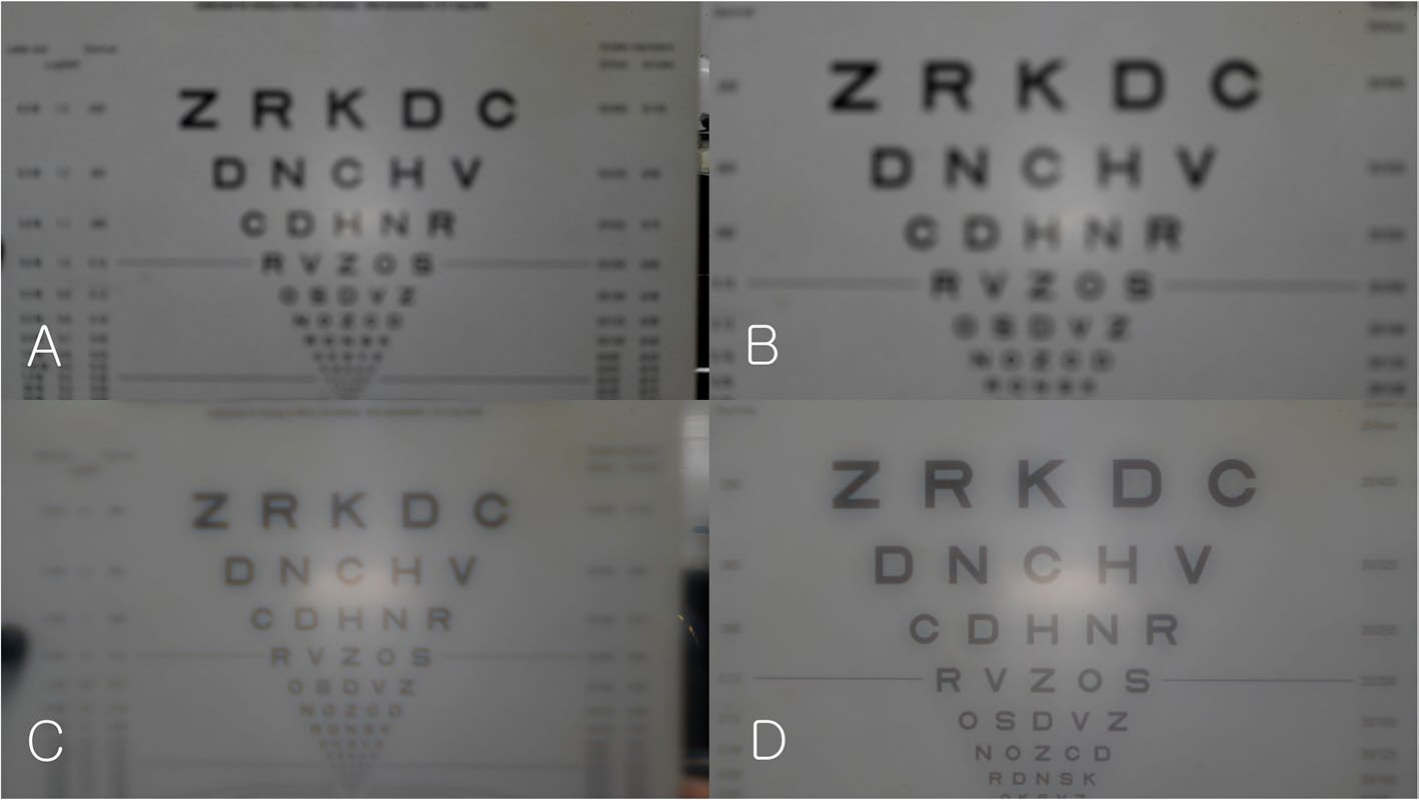
Digital single-lens reflex (DSLR) camera test (Near distance). With the monofocal lens, as the distance from the Early Treatment Diabetic Retinopathy Study (ETDRS) chart decreases, the images become increasingly blurry. With a multifocal lens, as the distance from the ETDRS chart decreases, they become very clear at a certain position. At this position, the distance between the pupil and ETDRS chart was approximately 500 mm. As the distance becomes smaller, it becomes blurry again. **A** Monofocal lens (the distance between the achromatic converging lens surface and the DSLR camera sensor: 660 mm); **B** Monofocal lens (distance: 500 mm); **C** Multifocal lens (distance: 660 mm); **D** Multifocal lens (distance: 500 mm)

Figure 13 and Videos 1, 2 show near-target images recorded with a monofocal lens and a multifocal lens. With the monofocal lens, as the distance from the Early Treatment Diabetic Retinopathy Study (ETDRS) chart decreases, the images become increasingly blurry (Video 1). With a multifocal lens, as the distance from the ETDRS chart decreases, they become very clear at a certain position. At this position, the distance between the pupil and ETDRS chart was approximately 500 mm. As the distance becomes smaller, it becomes blurry again (Video 2).

#### Far and near distance

**Fig. 14:**
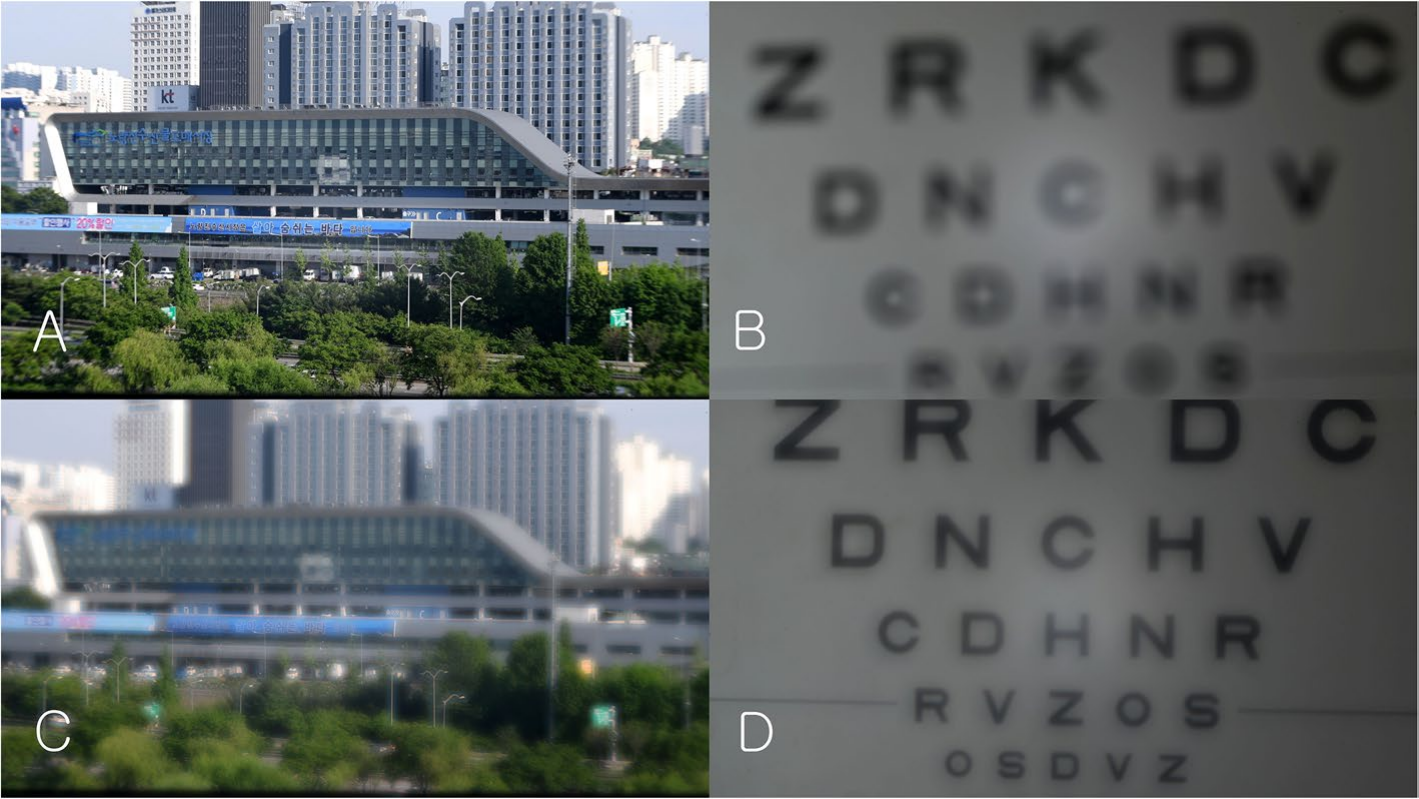
Digital single-lens reflex (DSLR) camera test (Far and near distance). With the monofocal lens, the building appeared very clear (**A**) but the letters in the ETDRS chart were very blurry at a distance of approximately 500 mm (**B**). With the multifocal lens, the building appeared slightly blurry (**C**) compared with the image from the monofocal lens, but the letters in the ETDRS chart appeared very clear (**D**) at a distance of approximately 500 mm

To test the multifocal function in the daytime, the ETDRS chart at near distance was recorded as a video right after recording a distant building (Fig. 14). With the monofocal lens, the building appeared very clear but the letters in the ETDRS chart were very blurry at a distance of approximately 500 mm (Fig. 14 A, B, Video 3). With the multifocal lens, the building appeared slightly blurry compared with the image from the monofocal lens, but the letters in the ETDRS chart appeared very clear at a distance of approximately 500 mm (Fig. 14 C, D, Video 4).

## Discussion

In this study, we clearly confirmed the multifocal function of a new multifocal lens created by combining a PDF lens and a converging lens. As expected, the multifocal lens has the properties of a bifocal lens focusing at 2 points. This multifocal lens, which combines a focal length +/− 100 mm (+/− 10 D) PDF lens and a focal length + 25 mm (+ 40 D) achromatic lens, produced a clear image at 0 and – 20 D defocus because the add power of the multifocal lens is 20 D. The images at undesired focal planes may be an indicator of stray light or leakage effects analogous to unwanted diffraction orders, possibly due to the less than 100% diffraction efficiency or the less than 50% energy going to the desired planes. In the optical bench test, the image at the distant focus of the multifocal lens (cross-correlation coefficient 0.878) was slightly blurry compared with the in-focus image of the monofocal lens (cross-correlation coefficient 1.0 by definition). This was similar to when the distant building was photographed with the DSLR camera using the multifocal lens.

At near distance, the object appeared very clear. In the optical bench test, the image at the near focus of the multifocal lens (cross-correlation coefficient 0.863) was clear. In the DSLR camera test, the ETDRS chart at a distance of approximately 500 mm was very clear when the second focus was located at the sensor of the camera. This was also demonstrated when the ETDRS chart at near distance was recorded as a video immediately after recording a distant building. This is not because the focusing at far objects more than 6 m away was incomplete. The multifocal function of the lens produced a clear image of near objects.

The combination of PDF and converging lenses is a completely different principle from that of diffractive multifocal IOLs. Diffractive multifocal IOLs have a multifocal function due to the diffraction of light from the concentric diffractive pattern [1]. Similarly to diffractive IOLs, the PDF lens has concentric rings (Fig. 3), so centration of the lens is important for good function. In addition, when the PDF lens was combined with a converging lens, the results showed a definite multifocal function: far objects appeared slightly blurry compared with a monofocal lens. These findings are similar to those using diffractive multifocal IOLs [10–16].

In optical bench test, we used a converging f = 25 mm lens and a PDF lens with f = + /− 100 mm. The PDF lens with f = + /− 100 mm was commercially available with the longest focal length. In the DSLR test, we used focal length +/− 500 mm (+/− 2 D) PDF lens (provided by Professor Seok Ho Song). Combining this PDF lens with an achromatic lens (focal length 100 mm, + 10 D) results in a trifocal lens (+ 8, 10, 12 D). Because the multifocal lens was positioned so that the second focal point (+ 10 D) of the multifocal lens was located at the sensor of the camera, the add power of this multifocal lens is 2 D. Therefore, with a multifocal lens, as the distance from the ETDRS chart decreases, the image becomes very clear at 500 mm from the multifocal lens. This multifocal lens would be closer to the add power (+ 2 D ~ 4 D) of the real multifocal IOLs compared with the commercially available PDF lens (+ 20 D, Edmund Optics).

Originally, a PDF lens acts as an f > 0 converging lens for right circular polarized light and an f < 0 diverging lens for left circular polarized light. If a PDF lens acts only for circularly polarized light, it would not be possible to use it as a multifocal IOL. According to the manufacturer’s data about the PDF lens we used, however, it serves as a converging lens f > 0 for 50% incidental light and a diverging lens f < 0 for 50% incidental light for not only circularly polarized light but also unpolarized light (https://www.edmundoptics.com/globalassets/documents/polarization-directed-flat-lens-overview.pdf), and our experiment demonstrated this. It also has the same function even when incidental light is linearly polarized, just like when wearing sunglasses with polarizing lenses (https://www.edmundoptics.com/globalassets/documents/polarization-directed-flat-lens-overview.pdf).

The advantages of our new multifocal lens are, first, that near objects look very clear. This was verified in the optical bench tests and DSLR camera tests. In this study, however, we did not compare our results with those of existing diffractive IOLs. Second, there was no energy loss because light energy is used 50% at near distance and 50% at far distance. On the other hand, in the case of existing diffractive IOLs, the light energy loss is 15 to 20% [17]. This is a large advantage of the newly invented multifocal lens.

The disadvantages of our new multifocal lens are, first, that there is quite a bit of chromatic aberration. In the optical bench test, we used an achromatic lens, such as the Badal lens, and when using only a 31.8-mm achromatic lens as a control, there was no chromatic aberration, so the chromatic aberration is considered to be due to the PDF lens, which was confirmed in the optical bench test and when taking photos of a building in the daytime. It may be possible to reduce this chromatic aberration by intentionally adjusting the chromatic aberration of the converging lens. Second, some of the cell phones or tablet personal computers that are often used at near distances have circular polarized displays. If we watch a linearly polarized display with linearly polarized sunglasses, we cannot see the display depending on its direction. If the display is circularly polarized, however, it is visible from all directions. As mentioned above, when incidental light is linearly polarized, the PDF lens we used serves as a converging lens f > 0 for 50% incidental light and a diverging lens f < 0 for 50%. Therefore, there is no problem in seeing the display at near distance (Supplemental Fig. 2). There may be a problem, however, if the display is circularly polarized. If it is RHCP, the PDF lens acts as a converging lens f > 0, so there is no problem seeing the display at near distance. Conversely, if the display is LHCP, the PDF lens will act as a diverging lens f < 0, making it difficult to see the display at near distance. Therefore, if some displays are RHCP and some displays are LHCP, a multifocal intraocular lens containing a PDF lens will not allow the patients to see all displays clearly at near distance. As a result of examining 2 smartphones, the display of the Galaxy Z flip (Samsung, Suwon, Korea) was linearly polarized, and the display of the iPhone6 (Apple, Cupertino, CA) was RHCP. Third, the human cornea is the ocular structure most likely to cause a change in the polarization state because, as shown in numerous studies, the cornea is birefringent; upon passage through the cornea linearly or circularly polarized light generally becomes elliptically polarized [18]. Different people have different cornea birefringence, which could impact the 50/50 division of the near and distant focal points with the PDF. This could be a flaw in this multifocal lens. Fourth, for the commercially available PDF lens used in the study, theoretically, the energy at far distance and near distance cannot be arbitrarily adjusted, and light energy is used 50% at near distance and 50% at far distance. If it is made as a multifocal IOL, near vision will be satisfactory, but distant vision may be relatively unsatisfactory to some patients. Specialists in the field (Professor Seok Ho Song), however, think that energy distribution can be changed by adjusting the film thickness.

The PDF lens film may be attached to the front or back surface of a monofocal IOL. The PDF lens used in the present study was stiff, but the PDF lens is an essentially thin flat film in which photo-aligned geometric phase holograms are recorded. Therefore, the IOL beneath the film can be folded for insertion. The biocompatibility of the PDF lens with the human eye, however, is not established, and thus it cannot yet be commercialized. It may be possible to use it by inserting it into an existing monofocal IOL for biocompatibility. It is still difficult to apply this lens directly to the human eye, but we introduce this new multifocal lens as a lens with the potential to function as a multifocal IOL.

## Conclusions

In this study, we clearly confirmed the multifocal function of a new multifocal lens created by combining a PDF lens and a converging lens. It is still difficult to apply this lens directly to the human eye, but we introduce this new multifocal lens as a lens with the potential to function as a multifocal IOL.

## Supplementary Information

**Additional file 1.**

**Additional file 2.**

**Additional file 3.**

## Data Availability

The datasets used and/or analyzed during the current study are available from the corresponding author on reasonable request.

## Abbreviations

PDF: Polarization-directed flat
D: Diopter
IOL: Intraocular lens
ETDRS: Early Treatment Diabetic Retinopathy Study
DSLR: Digital single-lens reflex
LED: Light-emitting diode
CCD: Coupled device
ISO: International Organization for Standardization
