# Supplementary figures and images for: Development of a novel multifocal lens using a polarization directed flat lens: possible candidate for a multifocal intraocular lens
Source: BMC Ophthalmol. 2021 Dec 27;21:444. doi: 10.1186/s12886-021-02191-z (PMC8711202; doi:10.1186/s12886-021-02191-z)

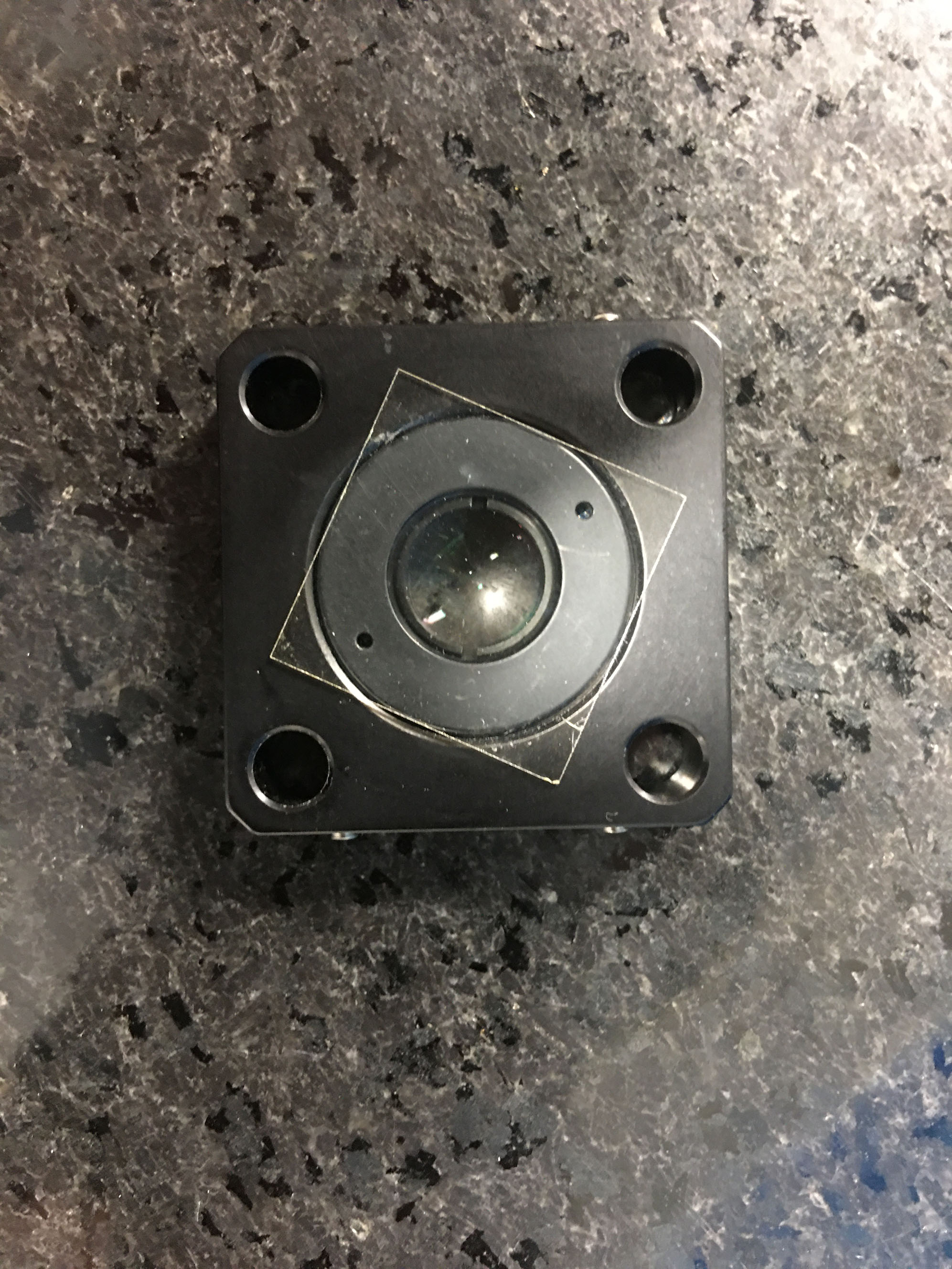

Supplement: Supplementary file 1 — Additional file 1. [file 12886_2021_2191_MOESM1_ESM.jpg]

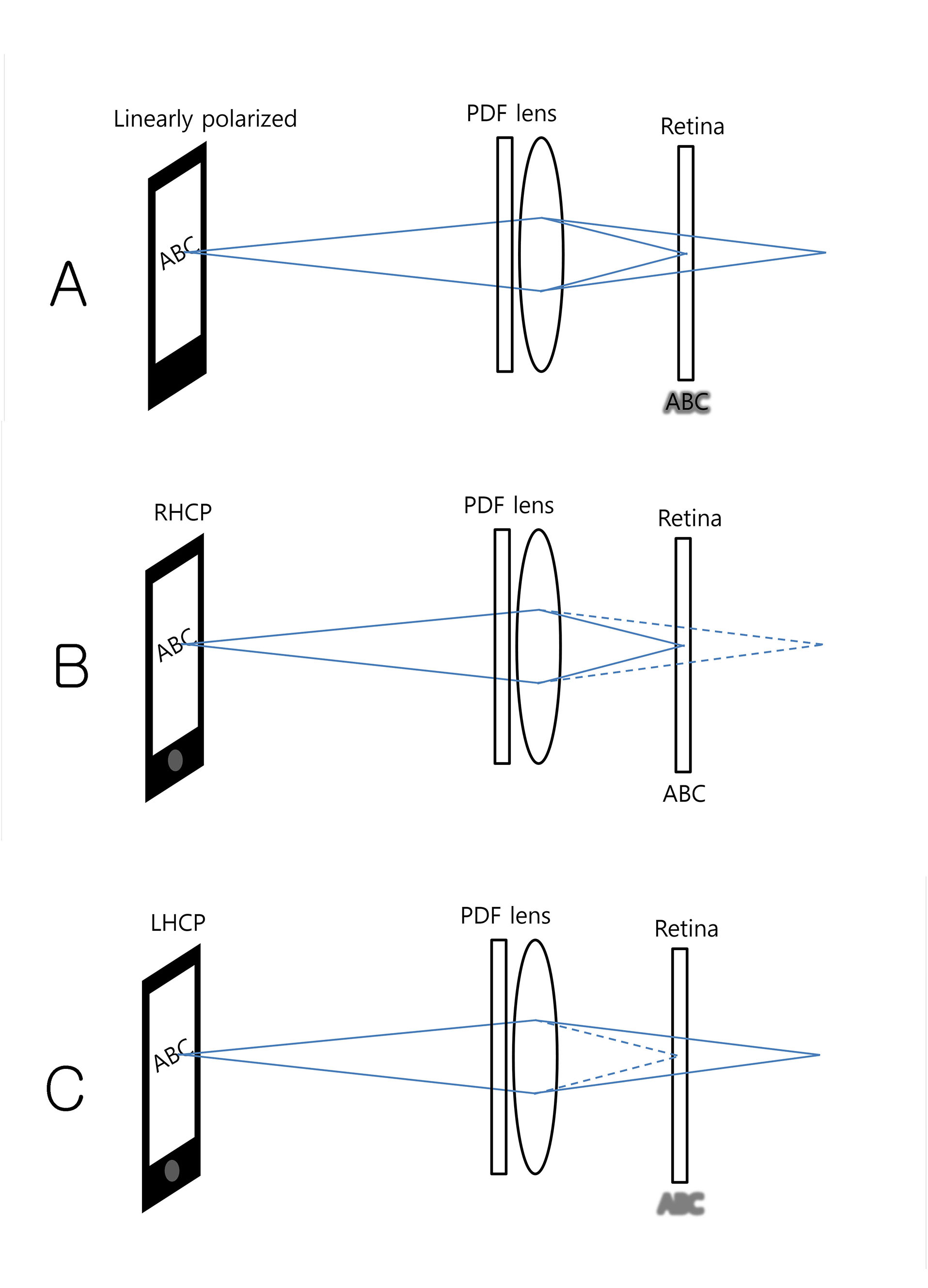

Supplement: Supplementary file 2 — Additional file 2. [file 12886_2021_2191_MOESM2_ESM.jpg]
