# Supplementary material for: Development of a novel multifocal lens using a polarization directed flat lens: possible candidate for a multifocal intraocular lens
Source: BMC Ophthalmol. 2021 Dec 27;21:444. doi: 10.1186/s12886-021-02191-z (PMC8711202; doi:10.1186/s12886-021-02191-z)
Supplement: Supplementary file 3 — Additional file 3. [file 12886_2021_2191_MOESM3_ESM.docx]

# **Supporting Information**

Supplement Figure 1. Polarization-directed flat (PDF) lens centration. For exact centration, we fixed the PDF lens on a 30-mm cage plate (Thorlabs Inc.) for the achromatic lens mount so that all four sides of the PDF lens contacted a center-located bore margin of it.

**Supplement Figure 2.** Polarization of display and polarization-directed flat (PDF) lens. When incidental light is linearly polarized, the PDF lens we used serves as a converging lens f >0 for 50% incidental light and a diverging lens f <0 for 50%. Therefore, there is no problem in seeing the display at near distance (A). There may be a problem, however, if the display is circularly polarized. If it is right-handed circular polarization (RHCP), the PDF lens acts as a converging lens f >0, so there is no problem seeing the display at near distance (B). Conversely, if the display is left-handed circular polarization (LHCP), the PDF lens will act as a diverging lens f < 0, making it difficult to see the display at near distance (C).

**Video 1.** Digital single-lens reflex (DSLR) camera test (Near distance, pupil diameter 4.0 mm). With the monofocal lens, as the distance to the ETDRS chart decreases, the images become increasingly blurry, but there was no chromatic aberration.

**Video 2.** Digital single-lens reflex (DSLR) camera test (Near distance, pupil diameter 4.0 mm). With a multifocal lens, as the distance from the ETDRS chart decreases, they become very clear at a certain position. At this position, the distance between the pupil and ETDRS chart was approximately 500 mm. Chromatic aberration is observed at the edges of the letters. As the distance becomes smaller, it becomes blurry again.

**Video 3.** Digital single-lens reflex (DSLR) camera test (Far and near distance, pupil diameter 4.0 mm). With the monofocal lens, the building appeared very clear but the letters in the ETDRS chart were very blurry at a distance of approximately 500 mm.

**Video 4.** DSLR camera test (Far and near distance, pupil diameter 4.0 mm). With the multifocal lens, the building appeared slightly blurry compared with the image from the monofocal lens, but the letters in the ETDRS chart appeared very clear at a distance of approximately 500 mm.
